# Prevalence of high-risk for obstructive sleep apnea in attention deficit hyperactivity disorder children referred to psychiatry clinic and impact on quality of life

**DOI:** 10.3389/fpsyt.2022.926153

**Published:** 2022-07-22

**Authors:** Tipkamol Prajsuchanai, Archwin Tanphaichitr, Tikumporn Hosiri, Kitirat Ungkanont, Wish Banhiran, Vannipa Vathanophas, David Gozal

**Affiliations:** ^1^Department of Otorhinolaryngology, Uthai Thani Hospital, Uthai Thani, Thailand; ^2^Department of Otorhinolaryngology, Faculty of Medicine Siriraj Hospital, Mahidol University, Bangkok, Thailand; ^3^Department of Psychiatry, Faculty of Medicine Siriraj Hospital, Mahidol University, Bangkok, Thailand; ^4^Department of Child Health and the Child Health Research Institute, University of Missouri School of Medicine, Columbia, MO, United States

**Keywords:** obstructive sleep apnea, sleep-disordered breathing, pediatric, questionnaire, attention deficit hyperactivity disorder, quality of life, Thailand

## Abstract

**Objectives:**

To study the prevalence of high-risk obstructive sleep apnea (OSA) in attention deficit hyperactivity disorder (ADHD) children in a child and adolescent psychiatry clinic using the Thai version of the Pediatric Obstructive Sleep Apnea Screening Tool (POSAST) questionnaire. The secondary objective was to evaluate the quality of life and identify associated factors for high-risk OSA in ADHD children.

**Study design:**

Prospective cross-sectional study.

**Material and method:**

Caregivers of pediatric patients aged 5–18 years old and diagnosed with ADHD by child and adolescent psychiatrists were surveyed about their child's sleeping habits.

**Results:**

Two hundred and seventy-four subjects were included. The patients' mean age was 10.4 ± 2.6 years, and 82.8% were males. There were 30 children (10.9%) diagnosed with obesity, 46 (16.8%) with chronic rhinitis, and 9 (3.3%) with asthma. The median duration of ADHD symptoms was 22.1 months. The prevalence of high-risk OSA was 18.2% and was associated with significantly reduced quality of life (adjusted OR = 4.46, 95% CI: 2.26–8.81, *P* < 0.001). A significant association between high-risk OSA and obesity also emerged (adjusted OR = 2.84, 95% CI: 1.17–6.88, *P* = 0.021).

**Conclusion:**

An elevated prevalence of high-risk OSA is present among Thai children with ADHD, and significantly impacts quality of life. A significant association between high-risk OSA and obesity is also detected in patients with ADHD. Therefore, screening for high-risk OSA in ADHD patients may likely facilitate early detection and treatment of OSA, and potentially prevent adverse consequences.

## Introduction

Obstructive sleep apnea (OSA) is a common disorder in children and is characterized by prolonged partial obstruction and intermittent complete obstruction of the upper airway that disrupts normal ventilation during sleep and sleep continuity. The prevalence of habitual snoring in children is 1.5–27.6% of the pediatric population and the prevalence of OSA is 1–5% (1). In Thailand, two studies reported a prevalence of habitual snoring at 4.3 and 8.5%, while the prevalence of OSA was 0.69, and 1.3% (2, 3). Among the major end-organ morbidities of OSA in children, cardiometabolic dysfunction and neurobehavioral alterations manifesting as poor academic performance and especially ADHD-like behaviors, have been ultimately associated with reduced quality of life. Studies examining the association between snoring, OSA, and ADHD have consistently identifies significant relationships (4–10). In addition, a meta-analysis confirmed that OSA and ADHD tend to co-exist (11), with 20–30% of ADHD children suffering from OSA (12). In Thailand, the prevalence of snoring in children with ADHD was 19.8% (13). However, the prevalence of high-risk OSA in ADHD children and the impact on quality of life have not been explored in Thailand. Despite the consensus that the gold standard for diagnosis of OSA is overnight polysomnography (PSG), there are limitations to the implementation of such approach in Thailand, including high costs, long waiting times, and the need for sleep technologists and sleep medicine physicians. Instead, an easy-to-use screening questionnaire for OSA could provide an alternative approach in resource-constrained environments such as in Thailand. To explore these possibilities, we undertook the current study based on the hypothesis that an elevated proportion of children diagnosed with ADHD would test positively, i.e., be at high-risk for OSA, when screened with a previously validated OSA questionnaire. As such, the primary objective of the study was to assess the prevalence of high-risk OSA in ADHD children by using the Thai version of the Pediatric Obstructive Sleep Apnea Screening Tool (POSAST) in a child and adolescent psychiatry clinic of a tertiary care hospital. A secondary objective was to evaluate OSA-specific quality of life by using the OSA-18 questionnaire in children diagnosed with ADHD, and to identify the associated factors for high-risk OSA in ADHD children.

## Materials and methods

### Sample size calculation

Based on the study by Silvestri et al. (14) which revealed an estimated prevalence of sleep-disordered breathing (SDB) in ADHD children of 21%, we used the 95% confidence intervals and derived the estimated cohort size using the equation:


$$\begin{array}{l} \text{n}=\frac{{\text{Z}}_{\alpha /2}^{2}\text{P}\left(1-\text{P}\right)}{{\text{d}}^{2}} \end{array}$$

*n* = number of subjects.

P = expected proportion = 0.21.

α = type I error = 0.05,

2-sided (95% Confidence Interval, Z = 1.96).

d = distance from proportion to limit = 0.05.


$$\begin{array}{l} n={\left(1.96\right)}^{2}\frac{\left(0.21\right)\left(1-0.21\right)}{{\left(0.05\right)}^{2}}\;=\;255 \end{array}$$

Then, we added another 10% for possible drop-offs and attrition during the survey, such that 280 subjects were deemed necessary.

### Study population

This study was performed after obtaining approval from Siriraj Institutional Review Board (SiRB), Protocol No. 379/2562(EC4). We consecutively recruited 280 subjects who were caregivers of pediatric patients aged 5–18 years old and diagnosed with ADHD in the child and adolescent psychiatry clinic of Siriraj Hospital, Bangkok, Thailand. The diagnosis of ADHD and other psychiatric comorbidities was conducted by child and adolescent psychiatrists using the Diagnostic and Statistical Manual of Mental Disorders, Fifth Edition (DSM-5) criteria (15). We ascertained that all caregivers could read the Thai language. Recruitment spanned the period from October 2019 to June 2020. Patients with a medical history of adenoidectomy, tonsillectomy, sinus surgery, Down syndrome, genetic syndromes, developmental delay, neuromuscular disorders, craniofacial anomalies, and chronic lung disease were excluded from the study.

### Study design

This study was a prospective cross-sectional study. Caregivers were surveyed about their child's sleeping habits using a previously validated questionnaire. In addition to demographic information and significant medical history of their child, the Thai Version of both POSAST and OSA-18 questionnaires were administered for evaluation of the risk for OSA and quality of life, respectively.

### Pediatric obstructive sleep apnea screening tool

There are several questionnaires used for OSA screening, but some have too many questions and unpredictable outcomes. Thus, this study was conducted by using a sensitive, short, and easy-to-use questionnaire named “Pediatric Obstructive Sleep Apnea Screening Tool (POSAST)” (16, 17). It is a validated 6-item questionnaire in which all questions are answered using a Likert-type response scale: “never” (0), “rarely” (once per week; 1), “occasionally” (twice per week; 2), “frequently” (three to four times per week; 3), and “almost always” (>4 times per week; 4), apart from the fifth question (i.e., mildly quiet = 0, medium loud = 1, loud = 2, very loud = 3, extremely loud = 4). The questions are Q1) Do you shake your child to breathe?, Q2) Have you witnessed an apnea during sleep?, Q3) Does your child struggle to breathe when asleep?, Q4) Do you have concerns about your child's breathing while asleep?, Q5) How loud does your child snore?, Q6) Does your child snore while asleep? The score derived from the POSAST is calculated as follows:

A = (Q1 + Q2)/2
B = (A + Q3)/2
C = (B + Q4)/2
D = (C + Q5)/2
Equation-derived score = (D + Q6)/2

A cut-off score of ≥ 1.9 rather than the original cut-off of ≥ 1.0 was used and indicative of high risk for the presence of moderate and severe OSA [correspond to an apnea-hypopnea index (AHI) ≥ 5 events per hour] according to the previously validated Thai version of the POSAST questionnaire (17, 18). A cut-off score of 1.9 yielded a sensitivity of 78.4%, a specificity of 50%, a positive predictive value of 76.3%, and a negative predictive value of 52.9%. In addition, we also implemented using the total additive score (Q1 + Q2 + Q3 + Q4 + Q5 + Q6) for diagnosing moderate and severe OSA [(AHI) ≥ 5 events per hour]. A cut-off of 8 yielded a sensitivity of 81.1%, a specificity of 52.8%, a positive predictive value of 77.9%, and a negative predictive value of 57.6% (18).

### Thai version quality of life questionnaire (OSA-18) for pediatric obstructive sleep apnea

The Thai version of the Quality of Life Questionnaire (OSA-18) for Pediatric Obstructive Sleep Apnea (19) was also used to assess the quality of life in this study. The OSA-18 consists of 18 items grouped in five domains of sleep disturbance (4 items), physical suffering (4 items), emotional distress (3 items), daytime problems (3 items), and caregiver concerns (4 items). Each item is scored in a seven-point Likert scale (1 = none of the time, 2 = hardly any of the time, 3 = a little of the time, 4 = some of the time, 5 = good amount of the time, 6 = most of the time, and 7 = all of the time). The total maximal score is 126 points. The decrease in quality of life was defined as ‘mild' if the score <60 points, “moderate” if the score was 60–80 points, and “severe” if the score > 80 points (20).

### Outcomes

The primary outcome was the prevalence of high-risk group for OSA in ADHD patients based on the scores obtained from the Thai version of POSAST, while a secondary outcome was the quality of life of the patients with ADHD who had high risk for OSA.

### Statistical analysis

Categorical data are presented as numbers and percentages. Continuous data are shown as mean ± standard deviation (SD) for normal distribution variables and median and interquartile range (IQR) for non-normal distribution variables. The Chi-Square test was used to compare the counts of categorical responses between two independent groups. Comparison of continuous data between groups was conducted by using unpaired *t*-tests for normal distribution variables and Mann-Whitney U test for non-normal distribution variables. A univariate analysis of associated factors for high risk OSA in ADHD children was performed. All variables with *P*-value <0.25 on univariate analysis were included in the multivariate analysis. All statistical analyses were performed using PASW Statistics version 18.0 (SPSS Inc, Chicago, Illinois). A *P*-value < 0.05 was considered statistically significant.

## Results

Two hundred and eighty caregivers of children diagnosed with ADHD were included in this study. Six subjects were excluded based on a history of previous adenotonsillectomy for symptoms of sleep disordered breathing. Therefore, 274 subjects were included in the final analyses. The patients' mean age was 10.4 ± 2.6 years, and they were predominantly males (82.8%). Overweight was present among 56 patients (20.5%), while 30 (10.9%) responders were obese. There were 46 patients (16.8%) diagnosed with chronic rhinitis and 9 children (3.3%) diagnosed with asthma. The median duration of ADHD was 22.1 (9.9, 45.5) months, and 49.6% had associated specific learning disorders. The demographic, nutritional status (21), and clinical characteristics of the patients are shown in Table 1. The prevalence of high-risk OSA was 18.2% when using the equation-derived score cut-off of 1.9 points, and 16.4% when using the total additive score cut-off of 8 points (Table 2).

**Table 1:** Demographic and clinical characteristics of the pediatric ADHD cohort (*n* = 274).

**Characteristic**	**Value**
Age, mean ± SD, year	10.4 ± 2.6
**Sex, *n* (%)**
Male	227 (82.8)
Female	47 (17.2)
**Nutritional status^a^, *n* (%)**	
Normal	188 (68.6)
Overweight	56 (20.5)
Obesity	30 (10.9)
**ADHD duration, median (IQR), months**	22.1 (9.89, 45.45)
**Associated psychiatric diseases, *n* (%)**	
Specific learning disorder	136 (49.6)
Oppositional defiant disorder	22 (8.0)
Autism spectrum disorder	5 (1.8)
Cognitive deficits	4 (1.5)
Vocal tic disorder	4 (1.5)
Anxiety disorder	4 (1.5)
Bipolar disorder	2 (0.7)
Obsessive compulsive disorder	2 (0.7)
**Psychiatric medication**	
Methylphenidate	211 (77.0)
Methylphenidate and anti-psychotics	47 (17.2)
Methylphenidate and fluoxetine	9 (3.3)
Methylphenidate and clonidine	5 (1.8)
No psychiatric medication	2 (0.7)

*ADHD, attention-deficit hyperactivity disorder; SD, standard deviation; IQR, interquartile range. ^a^Nutritional status was defined by weight for height normative reference values in Thai children. (21) Normal, % weight- for-height ≤ 120; overweight, % weight-for-height > 120–140; obesity, % weight-for-height > 140*.

**Table 2:** Prevalence of high-risk OSA in ADHD children (*n* = 274).

**OSA**	**Equation-derived score**		**Total additive score**	
	**Cut-off score**	**Prevalence**,	**Cut-off score**	**Prevalence**,
		***n*** **(%)**		***n*** **(%)**
Low risk	<1.9	224 (81.8)	<8	229 (83.6)
High risk	≥1.9	50 (18.2)	≥8	45 (16.4)

*OSA, obstructive sleep apnea; ADHD, attention-deficit hyperactivity disorder*.

Quality of life findings are shown in Table 3. Most of the participants (79.2%) reported mild decreases in quality of life, and 20.8% had moderate to severe reductions in quality of life. High-risk OSA among ADHD children was significantly associated with reduced quality of life (OR = 4.24, 95% CI: 2.18–8.25, *P* < 0.001; Table 4). Among 30 ADHD subjects with overweight/obesity, there were 10 children (33.3%) who had a high-risk for OSA. In addition, univariate analysis across multiple potential confounders revealed a significant association between high-risk OSA and obesity (OR = 2.55, 95% CI: 1.11–5.86, *P* = 0.023) as shown in Table 4. In the multivariate analysis, factor loading of variables with univariate association *P*-values <0.25, namely obesity, asthma, and quality of life revealed a significant independent association between high-risk OSA and obesity (adjusted OR = 2.84, 95% CI: 1.17–6.88, *P* = 0.021), and high-risk OSA and quality of life (adjusted OR = 4.46, 95% CI: 2.26–8.81, *P* < 0.001). Regarding asthma, a significant association emerged in the univariate analysis, but was not retained in the multivariate analysis (Table 5).

**Table 3:** Quality of life in ADHD children (*n* = 274).

**Impact on quality of life**	***n*** **(%)**
Mild (<60 points)	217 (79.2)
Moderate (60–80 points)	47 (17.2)
Severe (>80 points)	10 (3.6)

*ADHD, attention-deficit hyperactivity disorder*.

**Table 4:** Univariate analysis of associated factors for high-risk OSA in ADHD children (*n* = 274).

**Factors**	**Low Risk OSA, (*****n*** = **224)** ***n*** **(%)**	**High Risk OSA, (*****n*** = **50)** ***n*** **(%)**	**OR (95% CI)**	***P*** **-value**
Age, mean ± SD, year^a^	10.40 ± 2.61	10.36 ± 2.50	-	0.920
**Sex^b^**				0.810
Male	185 (82.6)	42 (84.0)	1	
Female	39 (17.4)	8 (16.0)	0.90 (0.39, 2.08)	
**Asthma^b^**				0.230
No	218 (97.3)	47 (94.0)	1	
Yes	6 (2.7)	3 (6.0)	2.32 (0.56, 9.61)	
**Chronic rhinitis^b^**				0.800
No	187 (83.5)	41 (82.0)	1	
Yes	37 (16.5)	9 (18.0)	1.11 (0.50, 2.48)	
**Obesity^b, d^**				0.023
No	204 (91.1)	40 (80.0)	1	
Yes	20 (8.9)	10 (20.0)	2.55 (1.11, 5.86)	
**ADHD duration**, median (IQR), month^c^	22.24 (9.61, 46.81)	21.39 (10.87, 36.83)	-	0.855
**Quality of life^e^**				<0.001
Mild	189 (84.4)	28 (56.0)	1	
Moderate to severe	35 (15.6)	22 (44.0)	4.24 (2.18, 8.25)	

*OSA, obstructive sleep apnea; ADHD, attention-deficit hyperactivity disorder; OR, odds ratio; CI, confidence interval; SD, standard deviation; IQR, interquartile range. The data was analyzed by using: ^a^Unpaired T-test, ^b^Chi-Square Test, ^c^Mann-Whitney U Test. ^d^Obesity was defined by % weight-for-height > 140. ^e^Impact on quality of life was defined as “mild” if the OSA-18 questionnaire score <60 points, and “moderate to severe” if the score > 60 points. Bold values indicate statistical significance*.

**Table 5:** Multivariate analysis of associated factors for high-risk OSA in ADHD children (*n* = 274).

**Factors**	**Adjusted OR (95% CI)**	***P*** **-value**
Obesity	2.84 (1.17, 6.88)	0.021
Asthma	2.49 (0.52, 11.87)	0.251
Quality of life	4.46 (2.26, 8.81)	<0.001

*OSA, obstructive sleep apnea; ADHD, attention-deficit hyperactivity disorder; CI, confidence interval. Bold values indicate statistical significance*.

## Discussion

Our study demonstrated a relatively elevated prevalence of high-risk OSA among Thai children diagnosed with ADHD (18.2%) by using the previously validated POSAST questionnaire. According to previous publications, the prevalence of OSA in community Thai children was estimated at 0.69–1.3% (2, 3). Several previous studies have identified an association between OSA and ADHD around the world (4, 11, 12). The current study has uncovered that the validated POSAST sleep questionnaire can be used as a screening tool to identify the children with high-risk for OSA in a pediatric psychiatry clinic. Accordingly, ADHD children at high-risk for OSA based on the POSAST instrument should be referred to specialists for further evaluation. Our findings are in close concordance with a systematic review that revealed an estimated prevalence of OSA in ADHD children between 20 and 30% (12). Although the mechanisms underlying the increased prevalence of OSA among children with ADHD remain unknown, previous work in rodent models (22, 23) has led to the assumption that intermittent hypoxia and disrupted sleep induced by upper airway dysfunction during sleep might impose an adverse impact on brain structure and function (24–26), as well as on cognitive function (5, 27, 28), leading to inattention and hyperactivity in developing subjects (5, 28, 29). The presence of high-risk OSA was also associated with an increased risk for reduced quality of life, similar to the findings reported by a meta-analysis by Baldassari et al. (30) From the results of 10 separate studies, 3 studies compared the quality of life in children with OSA and healthy children using the Child Health Questionnaire (CHQ), and found that children with OSA had poorer quality of life. In the other 7 publications, 369 children with OSA undergoing adenotonsillectomy were evaluated using the OSA-18 questionnaire. The total OSA-18 score and each of the domain scores showed significant improvements after adenotonsillectomy and remained improved during long-term follow-up (30). Furthermore, in the only randomized controlled study to date, the CHAT study, significant improvements in quality of life emerged in the group undergoing adenotonsillectomy when compared to the group assigned to watchful waiting (31). Since the prevalence of high-risk OSA in children with ADHD was high and significantly associated with reduced quality of life, screening for OSA in ADHD patients is recommended. Screening for pediatric OSA in children with ADHD or other risk groups by using a questionnaire such as POSAST is easy and cost-effective.

Similar to previous studies (32, 33), the current study uncovered a significant association between OSA and obesity. We found the prevalence of high-risk OSA in obese children was 33.3%. This result was in accordance with the previous study with the prevalence of OSA among obese children was 44.6% (32, 33). We should also point out that children suffering from ADHD are also at higher risk of being overweight or obese. The odds ratio for obesity in ADHD children with high-risk OSA was 2.55, which is similar to the previous study that demonstrated the odds ratio for obesity in children with OSA was 4.69 (32, 33). Moreover, the presence of somnolence in children with ADHD may be facilitated by the underlying presence of concurrent obesity and OSA (34, 35).

Several studies have explored the potential relationships between asthma and sleep-disordered breathing. Most of the studies have uncovered a substantial risk afforded by the presence of asthma on OSA-related risk (36–38). In a large multicentric cross-sectional study involving 22,478 children aged 5–12 years, the authors reported that the prevalence of SDB and asthma were 12 and 3.5%, respectively and that habitual snoring and OSA were significantly associated with asthma with corresponding odds ratios of 1.28 and 1.92 (39). Such findings are remarkably similar to the current study. However, although the association between asthma and OSA in patients with ADHD was statistically significant in the univariate analysis, it did not persist in the multivariate analysis, possibly due to the relatively small number of patients diagnosed with asthma in our study.

There were limitations in this study. First, the diagnosis of OSA in this study was done by using POSAST, which is considered a subjective tool. Because of limitations of the resources for using PSG, which is the gold standard for the diagnosis of OSA, we considered using the questionnaire as a screening tool for identifying patients with high risk for moderate and severe OSA. Then, these patients should be referred to specialists for further management. Second, the risk for OSA in patients who had a POSAST score less than the cut-off could not be ruled out. Follow-up is recommended and if there are persistent symptoms or signs suggestive of OSA, the patients should also be referred to specialists for further evaluation. Third, this study did not include patients with specific underlying conditions who are at high risk for OSA, such as Down syndrome, craniofacial anomalies, or neuromuscular diseases. We also included only pediatric patients aged 5 years and older in this study because of the difficulty for diagnosis of ADHD in a younger age group. Hence, a high index of suspicion of OSA in ADHD patients at a younger age is recommended.

## Conclusion

The prevalence of high-risk OSA in children with ADHD is high and appears to impose a significant detrimental effect on the quality of life. Therefore, screening for OSA among ADHD patients is recommended and should enable early detection along with timely treatment ultimately aiming at the prevention of the adverse consequences of OSA.

## Data availability statement

The datasets presented in this article are not readily available because no potential identifiable data was provided. Requests to access the datasets should be directed to archwin.tan@mahidol.ac.th.

## Ethics statement

The studies involving human participants were reviewed and approved by Siriraj Institutional Review Board, Faculty of Medicine Siriraj Hospital, Mahidol University. The patients/participants provided their written informed consent to participate in this study.

## Author contributions

TP, AT, and TH contributed to conception and design of the study. TP organized the database and wrote the first draft of the manuscript. TP and AT performed the statistical analysis. AT, KU, and DG contributed to manuscript revision. All authors contributed to read and approved the submitted version.

## Funding

This research project was supported by Siriraj Research Fund, grant number (IO) R016231063, Faculty of Medicine Siriraj Hospital, Mahidol University. DG was supported by the Leda J Sears Foundation.

## Conflict of interest

The authors declare that the research was conducted in the absence of any commercial or financial relationships that could be construed as a potential conflict of interest.

## Publisher's note

All claims expressed in this article are solely those of the authors and do not necessarily represent those of their affiliated organizations, or those of the publisher, the editors and the reviewers. Any product that may be evaluated in this article, or claim that may be made by its manufacturer, is not guaranteed or endorsed by the publisher.
